# A Real-Time Mobile Intervention to Reduce Sedentary Behavior Before and After Cancer Surgery: Usability and Feasibility Study

**DOI:** 10.2196/17292

**Published:** 2020-03-23

**Authors:** Carissa A Low, Michaela Danko, Krina C Durica, Abhineeth Reddy Kunta, Raghu Mulukutla, Yiyi Ren, David L Bartlett, Dana H Bovbjerg, Anind K Dey, John M Jakicic

**Affiliations:** 1 University of Pittsburgh Pittsburgh, PA United States; 2 Carnegie Mellon University Pittsburgh, PA United States; 3 University of Washington Seattle, WA United States

**Keywords:** sedentary behavior, mobile health, smartphone, mobile phone, wearable device, surgical oncology, physical activity

## Abstract

**Background:**

Sedentary behavior (SB) is common after cancer surgery and may negatively affect recovery and quality of life, but postoperative symptoms such as pain can be a significant barrier to patients achieving recommended physical activity levels. We conducted a single-arm pilot trial evaluating the usability and acceptability of a real-time mobile intervention that detects prolonged SB in the perioperative period and delivers prompts to walk that are tailored to daily self-reported symptom burden.

**Objective:**

The aim of this study is to develop and test a mobile technology-supported intervention to reduce SB before and after cancer surgery, and to evaluate the usability and feasibility of the intervention.

**Methods:**

A total of 15 patients scheduled for abdominal cancer surgery consented to the study, which involved using a Fitbit smartwatch with a companion smartphone app across the perioperative period (from a minimum of 2 weeks before surgery to 30 days postdischarge). Participants received prompts to walk after any SB that exceeded a prespecified threshold, which varied from day to day based on patient-reported symptom severity. Participants also completed weekly semistructured interviews to collect information on usability, acceptability, and experience using the app and smartphone; in addition, smartwatch logs were examined to assess participant study compliance.

**Results:**

Of eligible patients approached, 79% (15/19) agreed to participate. Attrition was low (1/15, 7%) and due to poor health and prolonged hospitalization. Participants rated (0-100) the smartphone and smartwatch apps as very easy (mean 92.3 and 93.2, respectively) and pleasant to use (mean 93.0 and 93.2, respectively). Overall satisfaction with the whole system was 89.9, and the mean System Usability Scale score was 83.8 out of 100. Overall compliance with symptom reporting was 51% (469/927 days), decreasing significantly from before surgery (264/364, 73%) to inpatient recovery (32/143, 22%) and postdischarge (173/420, 41%). Overall Fitbit compliance was 70% (653/927 days) but also declined from before surgery (330/364, 91%) to inpatient (51/143, 36%) and postdischarge (272/420, 65%).

**Conclusions:**

Perioperative patients with cancer were willing to use a smartwatch- and smartphone-based real-time intervention to reduce SB, and they rated the apps as very easy and pleasant to use. Compliance with the intervention declined significantly after surgery. The effects of the intervention on postoperative activity patterns, recovery, and quality of life will be evaluated in an ongoing randomized trial.

## Introduction

Surgery is the first step of curative treatment for most cancers, but despite advances in surgical techniques and perioperative care, postoperative morbidity and complication rates remain high. Risks are particularly high after advanced abdominal cancer resection and include 30%-40% major complication rates, 15%-40% readmission rates, 40% reduction in functional capacity, and significant persistent symptoms [1-3]. Supportive behavioral interventions to enhance postoperative functioning and reduce risks of complication and readmission are needed.

Perioperative physical activity is a promising target for behavioral intervention given evidence that higher step counts after cancer resection are associated with lower readmission risk [4]. In the context of major abdominal cancer surgery, breaking up prolonged sedentary behavior (SB) bouts with brief walking breaks may be more attainable than increasing moderate physical activity (PA) or aiming for a specific step count goal. SB, defined as low energy expenditure activity in a seated or reclined position during waking hours, shows a sustained and marked increase after gastrointestinal cancer surgery, with patients spending more than 95% of their time sitting or lying in the week after surgery [5]. Prolonged SB after surgery could lead to physical deconditioning and reduced functional capacity that increases short- and long-term risks [6]. Independent of the health protective effects of moderate-to-vigorous PA, excessive SB has also been associated with lower quality of life and increased mortality in cancer survivors [7-9].

The growing ubiquity of smartphones and wearable activity monitors offers an unprecedented opportunity to harness real-time SB data and to deliver behavioral interventions before surgery, during inpatient recovery, and after hospital discharge as patients recover at home. Mobile technology is increasingly being utilized to deliver PA interventions, with emerging data suggesting that mobile health interventions can effectively increase PA [10,11] and are acceptable for patients with cancer and survivors [12,13]. Given evidence that physical symptoms are the primary barrier to breaking up SB in patients with cancer and survivors [14,15], mobile technology can also be used to collect patient-reported symptom data that can be leveraged to tailor recommendations to be responsive to fluctuations in health. The goal of this pilot study was to develop and test a mobile technology–supported intervention to reduce SB before and after cancer surgery. We conducted a single-arm pilot trial evaluating the usability and acceptability of a real-time mobile intervention that detects prolonged SB in the perioperative period and delivers prompts to walk that are tailored to daily self-reported symptom burden.

## Methods

### Participants

Participants were recruited between June and September 2018 at their preoperative clinic visit. Potential research participants were identified by their surgical oncology care team, who confirmed eligibility. If patients expressed interest in learning more about the study, they were approached by the research team after consenting to and scheduling surgical treatment of metastatic colorectal or peritoneal cancer. The study was open to English-speaking adults able to stand and walk unassisted. Patients who were less than 2 weeks from their scheduled surgery date were excluded, which ensured that participants had adequate time to become familiar with the study’s technology and activity prompts prior to surgery.

### Study Procedures

After providing written informed consent, participants completed a questionnaire to collect information about demographic variables, health behaviors, and experience with mobile technology. They were provided with a Fitbit Versa smartwatch paired with a Google Pixel 2 smartphone on which the Detecting Activity to Support Healing (DASH) study app as well as the Fitbit app had been installed. From the time of consent to 30 days after hospital discharge following their surgery, participants were asked to keep the devices charged, to wear the smartwatch as much as possible, to rate their daily experience of symptom severity once each morning, and to respond to activity prompts. Participants were called once per week, when feasible, to complete semi-structured interviews about their experiences with the intervention. A questionnaire about the usability of the apps was administered at the end of the study. All procedures were approved by the University of Pittsburgh Institutional Review Board.

### The DASH Study App and Intervention

**Figure 1:**
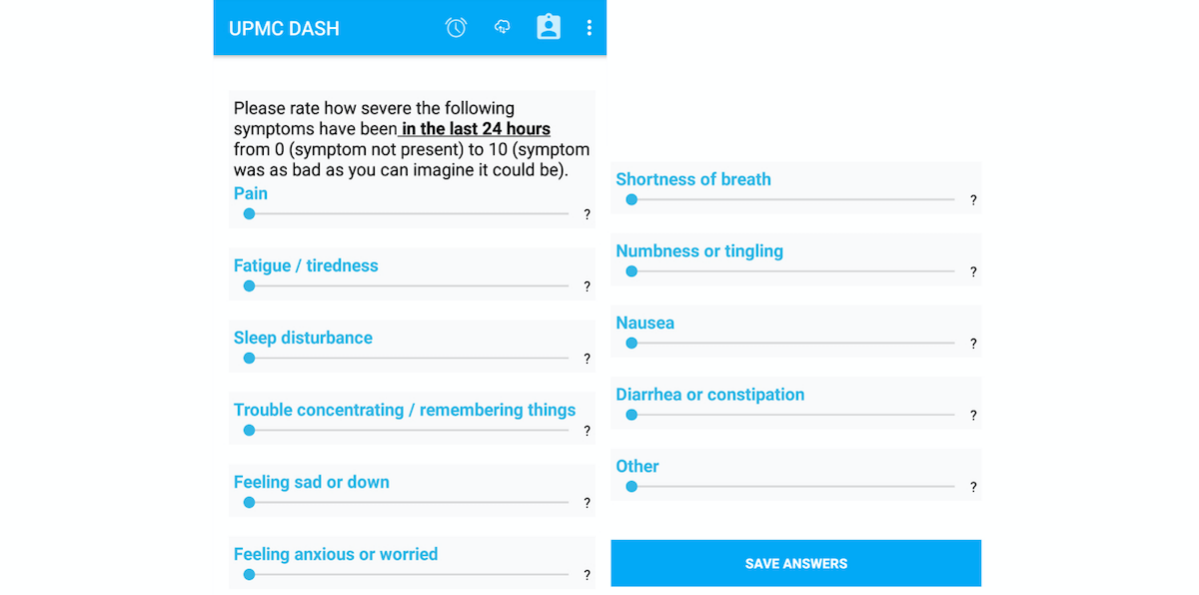
Daily symptom severity rating on smartphone app. DASH: Detecting Activity to Support Healing.

The DASH Study Android smartphone app, created by members of the research team, sent a notification to participants each morning (at a time that was set and could be adjusted by participants) reminding them to rate the severity of 10 symptoms (pain, fatigue/tiredness, sleep disturbance, trouble concentrating/ remembering things, feeling sad or down, feeling anxious or worried, shortness of breath, numbness or tingling, nausea, and diarrhea or constipation) they had experienced in the last 24 hours using a scale from 0 (symptom not present) to 10 (symptom as bad as you can imagine; Figure 1).

**Figure 2:**
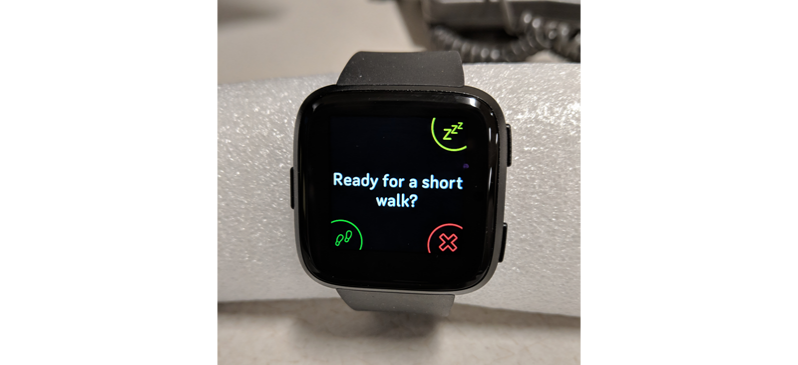
Activity prompt on Fitbit Versa smartwatch app.

**Figure 3:**
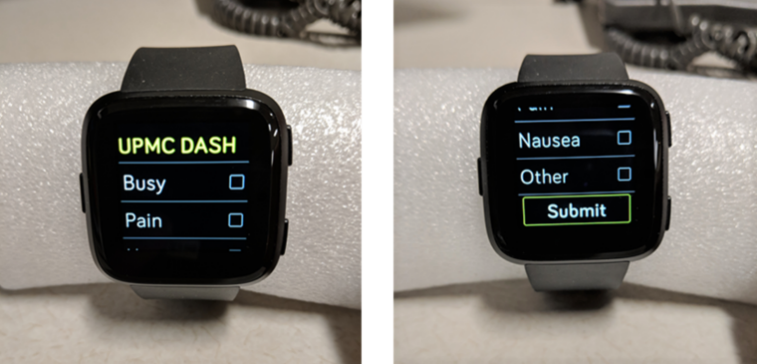
If responding “No”, provide reason(s) you are unable to walk. DASH: Detecting Activity to Support Healing.

The DASH Study Fitbit OS smartwatch app used this information to set a threshold for SB bouts and used real-time step count data to trigger activity prompt notifications when that threshold was exceeded. If a morning symptom rating was not completed, the most recent symptom rating was carried forward to select that day’s SB threshold. Activity prompts were sent when: (1) SB (defined for the purposes of this study as fewer than 50 cumulative steps since the most recent activity prompt) exceeded 60 consecutive minutes, and at the most recently completed symptom rating, all symptoms were rated less than 7 out of 10 or (2) SB exceeded 120 consecutive minutes and any symptom was rated 7 or higher. When SB thresholds were exceeded, an activity prompt notification (“Ready for a short walk?”) was sent to both the smartphone and the smartwatch (Figure 2). Participants could respond on either the watch or the phone with the response options Yes, No, or Snooze. If Snooze was selected, an activity prompt was sent again 15 minutes later. If No was selected, participants were asked to indicate their reason(s) for not walking (Busy, Pain, Nausea, or Other; Figure 3). Regardless of response, participants received a positive feedback message (“Great job being active!”) if 30 or more steps were logged within 15 minutes of an activity prompt. Daily step counts as well as sleep data were also available to view in the Fitbit app as desired.

### Measures

Usability was assessed in two ways: (1) via weekly ratings on a scale of 0 to 100 on how easy it was to use the smartphone and smartwatch apps; how pleasant the interface of each app was in terms of appearance, design, and usability; and how satisfied the participant was overall with the DASH intervention and (2) via the System Usability Scale, a widely used ten-item questionnaire used to evaluate technological systems that was administered at the end of the intervention [16]. Notes from the semi-structured interviews were also reviewed by the research team and organized into identified themes related to issues encountered, suggestions for improvement, and other feedback.

Feasibility was assessed via accrual and retention rates as well as compliance with reporting symptoms. Objective activity and heart rate data indicated compliance with wearing the smartwatch as well as walking in response to activity prompts. Daily compliance with wearing the watch was defined as logging at least some activity (more than 0 steps) or heart rate data (more than 0 beats per minute).

## Results

### Participant Characteristics

Table 1 shows that the sample was primarily female, white, well-educated, and familiar with technology. Participants started using the mobile health intervention an average of 26 days (range 11-40) prior to surgery, throughout their inpatient stay (mean 10.4 days, range 6-15 days), and for 30 days postdischarge, for an average of 66 total days (range 47-81) using the intervention.

**Table 1:** Participant characteristics (N=15).

Characteristics		Value
Age (years), mean (range)		49.7 (25-65)
**Sex, n (%)**		
	Female	12 (80)
	Male	3 (20)
**Race, n (%)**		
	White	13 (87)
	Black	2 (13)
**Marital status, n (%)**		
	Married	9 (60)
	Divorced/separated/widowed	4 (27)
	Never married	2 (13)
**Employment status, n (%)**		
	Working full-time	6 (40)
	Working part-time	2 (13)
	Retired/not working	7 (47)
**Education, n (%)**		
	High school diploma or equivalent	4 (27)
	Some college	5 (33)
	Bachelor’s degree or higher	6 (40)
Body mass index, mean (SD)		27.2 (6.4)
**Smoking history, n (%)**		
	Current smoker	2 (13)
	Former smoker	8 (53)
	Never smoker	5 (33)
**Exercise frequency, n (%)**		
	Seldom or never	5 (33)
	1-2 times per week	4 (27)
	3-4 times per week	3 (20)
	5 or more times per week	3 (20)
Has Wi-Fi at home, n (%)		14 (93)
Owns a computer, n (%)		13 (87)
Owns a tablet, n (%)		10 (67)
Owns a smartphone, n (%)		14 (93)
Owns an activity tracker, n (%)		1 (7)
Uses social media, n (%)		12 (80)

### Usability

On a scale from 0 to 100, participants rated the smartphone and smartwatch apps as very easy (mean 92.3 and 93.2, respectively) and pleasant to use (mean 93.0 and 93.2, respectively). Overall participant satisfaction with the whole system was 89.9, and the mean System Usability Scale score at the end of the study was 83.8 (maximum possible score of 100).

Overall, participants reported that the activity prompts were mostly sent at an appropriate frequency and that they liked the simple wording of the prompts. Some participants found the Fitbit Versa to be bulky and unattractive, reported that the smartwatch did not seem to accurately record all steps (especially when walking slowly or with assistance), and reported occasional syncing or connectivity issues between the watch and the phone. The primary complaint about the smartphone app was that the slider used to adjust the symptom rating did not always work smoothly. Participants were generally satisfied with the “No” response options but wished they could elaborate on the “Busy” or “Other” responses on the watch. Participants also reported that it was especially difficult to walk in the hospital immediately after surgery when they were too weak to walk unassisted, were in the middle of tests or other care procedures, or were on medications that made it difficult to get up and walk. One participant (a 40-year-old white woman with a preoperative exercise frequency of 1-2 times per week) said, “During the hospital was the toughest part; you don't even want to talk to anybody or even think about technology...devices were the last thing (I) wanted to worry about.”

At the end of the study, multiple participants reported that the system was motivating. One participant (a 55-year-old white man with a preoperative exercise frequency of 1-2 times per week) said, “It’s cool to track how many steps I have and see what days were good days and what days were bad. It helps motivate (me) to walk.” Another participant (a 51-year-old white female with a preoperative exercise frequency of 3-4 times per week) said that the smartwatch “made (me) more conscious of the need to move, before and after surgery.” One participant (a 25-year-old black woman with a preoperative exercise frequency of 3-4 times per week) commented that she wished the system had been more personalized, because it was so simple and “felt generic.”

### Feasibility

Of the 19 eligible patients approached, 15 agreed to participate (79% accrual rate). Reasons for not participating were “too busy/overwhelmed” (n=2) and “not good with technology” (n=2). The retention rate for the study was 93% (14/15), and the 1 patient who did not complete the study withdrew due to poor health and prolonged hospitalization.

Over the course of the study, daily symptom ratings were completed 51% (469/927) of the days, with compliance rates decreasing significantly from before surgery to inpatient recovery and postdischarge (Table 2). Across all days that symptoms were rated, 37% (172/469) were classified as a high symptom day and were accompanied by a higher SB threshold, and the most common symptoms rated as severe (≥7 out of 10) were fatigue and pain. The frequency of severe symptom days increased slightly from the presurgery waiting period to postoperative inpatient recovery.

**Table 2:** Trends in compliance, symptoms, and activity over the perioperative course.

Variable	Preoperative	Inpatient recovery	Postdischarge
Symptom reporting compliance, % (n/N)	73 (264/364)	29 (42/143)	41 (173/420)
Severe symptom days, % (n/N)	34 (90/264)	47 (15/32)	39 (67/173)
Smartwatch wearing compliance, % (n/N)	91 (330/364)	36 (51/143)	65 (272/420)
Daily step count, mean (SD, range)	5865 (3113, 637-21,115)	1594 (1567, 0-6100)	2054 (1753, 42-9645)
Average sedentary behavior bout duration (minutes), mean (SD, range)	23 (12, 7-94)	177 (201, 32-720)	72 (72, 17-720)

Step count data were collected on 70% (653/927) of the days, but compliance with wearing the smartwatch also declined from before surgery to inpatient and postdischarge. On average, participants logged 3944 steps per day (SD 3185, range 0-21,115) with an average SB bout duration of 55 minutes (range 7-720, SD 83). As expected, step counts decreased significantly and average SB bout durations increased significantly from before surgery to during inpatient recovery (Table 2), but an important limitation is that these mean step counts and SB bout values are based on the subset of participants who were compliant with wearing the smartwatch.

Unfortunately, due to data syncing issues, timestamped logs of all activity prompts and participant responses were not available for all participants during this pilot deployment. Activity prompts and participant responses were completely or partially logged for 8 participants. For these participants, an average of 133 activity prompts were sent during the deployment for an average of 4.18 prompts per day when activity prompts were logged. Overall participants walked and received positive feedback messages after 27% (288/1064) of the prompts, although walking was detected after only 14% (45/311) of the prompts sent during inpatient recovery.

Participant age and gender were not significantly related to pleasantness or ease of use ratings, System Usability Scores, or smartwatch wearing compliance. Older age was significantly correlated with higher symptom rating compliance (*r*_14_=.61, *P*=.02).

## Discussion

### Principal Findings

This study describes the successful development and preliminary testing of a mobile technology-based intervention to reduce SB before and after abdominal cancer surgery, with recommendations tailored to patient-reported symptom severity. Perioperative patients with cancer were willing to use a smartwatch- and smartphone-based intervention to reduce SB in real time, and they rated the apps as very easy and pleasant to use. Participants generally reported that they liked the simplicity of the intervention and found the prompts to be motivating. However, overall compliance with completing daily symptom ratings, wearing the smartwatch, and walking after receiving an activity prompt declined significantly from before to after surgery, and compliance with symptom reporting did not significantly improve even after patients were discharged from the hospital. This significant drop in engagement with the intervention after surgery may limit the effects of this behavioral intervention on postoperative outcomes.

The high usability ratings and low postoperative compliance rates observed in our study are consistent with previous work testing mobile health apps in gastrointestinal surgery patients. In one study testing a symptom, wound, and temperature tracker after colorectal surgery, participants rated the app as highly usable, but 30% of participants never used the app and 10% used the app only once [17]. In a study testing a similar symptom-monitoring app along with Fitbit monitoring and hydration reminders, 89% of patients described the app as easy to use, with Fitbit data collected on 85% of days, but only 68% completed symptom ratings and 51% uploaded photos of their wounds [18]. These studies were not attempting to modify patient activity behavior, but the barriers to daily use of the mobile apps after surgery reported in those studies (eg, postoperative pain and fatigue or trouble remembering to use the app if it was not part of a typical routine) are likely to be similar to those in our study and should be carefully considered when designing mobile apps for perioperative patients with cancer.

The declining compliance in Fitbit wear time is also consistent with studies in healthy adults, which showed that 40% of volunteers abandoned the Fitbit within six months [19]. Of note, there was significant variability in postoperative compliance, with some participants maintaining high levels of engagement before and after surgery and others disengaging completely after surgery. Decreases in compliance were particularly marked in the 43% (6/14) of patients who were readmitted within 30 days. Finding ways to maintain patient engagement during inpatient recovery and beyond is an important future goal for this research. These strategies may include contacting patients more frequently or involving caregivers in the intervention to emphasize feelings of being cared for and monitored [20]. Given that very few activity prompts (45/311, 14%) delivered to patients while in the hospital were followed by walking breaks, the frequency of notifications may have been too often or the definition of a walking break (30 steps in 15 minutes) not attainable during this time of acute illness; therefore, increasing the threshold of SB permitted during hospitalization could also be useful.

The intervention was designed to be responsive to daily patient-reported symptom burden based on the hypothesis that increasing the threshold for SB on days with even 1 severe symptom may make the intervention more attainable for patients, resulting in better self-efficacy. However, low compliance with symptom reporting after surgery limited the ability of the intervention algorithm to reduce the frequency of activity prompts on such days. Only 47% of days (15/32) with symptom data available during inpatient recovery were classified as including one or more severe symptoms, possibly because participants were more likely to be compliant with symptom reporting on days when they were feeling better and less symptomatic. Estimation of high symptom burden based on passive sensors within the smartphone and smartwatch, which could be done with minimal burden to patients, is another important direction for future research [21].

To our knowledge, this is the first study to use both patient-reported symptoms and real-time activity monitoring data to deliver a SB intervention and the first study to explicitly target SB before and after cancer surgery. Strengths of the study included the use of real-time step count data to trigger activity prompts that were tailored to patient-reported symptom ratings and the use of ubiquitous commercial devices to deliver the intervention.

### Limitations

This study also had several limitations. The sample size for this feasibility study was small and was biased toward well-educated younger female patients, who may have been more willing to participate in a technology-supported behavior change intervention. All patients were scheduled for surgery for metastatic peritoneal cancer, and results may not generalize to other surgical oncology or surgery populations. Complete data about activity prompts delivered and participant responses to prompts were not available, limiting our ability to examine participant adherence to recommended walking breaks. The intervention used a study-provided Android smartphone, although 14 of 15 participants already owned a smartphone. The need to carry and charge a second device across perioperative transitions of care may have contributed to poor compliance to symptom reporting as well as syncing issues. Future studies should consider installing study apps on participants’ personal devices to minimize participant burden and increase the likelihood of participant engagement and compliance.

### Conclusions

In conclusion, mobile technology-based interventions have the potential to improve postoperative outcomes after cancer surgery by targeting modifiable behaviors in real-time. Results from this pilot study demonstrate moderate feasibility and acceptability and good usability of a real-time mobile technology–based SB intervention for perioperative patients with abdominal cancer. Future research involving perioperative mobile health interventions should consider ways to enhance postoperative compliance, including engaging caregivers or providing additional personalization of behavioral interventions. A randomized controlled trial testing preliminary effects of the intervention on postoperative activity patterns, recovery, and quality of life is currently underway.

## Abbreviations

DASH: Detecting Activity to Support Healing
PA: physical activity
SB: sedentary behavior
